# Supplementary material for: Higher skeletal muscle mitochondrial oxidative capacity is associated with preserved brain structure up to over a decade
Source: Nat Commun. 2024 Dec 30;15:10786. doi: 10.1038/s41467-024-55009-z (PMC11686348; doi:10.1038/s41467-024-55009-z)
Supplement: Supplementary file 1 — Supplementary Information [file 41467_2024_55009_MOESM1_ESM.pdf]

Supplementary Table 1. Longitudinal associations of skeletal muscle oxidative capacity with brain volume changes after adjusting for 400m time (n=627), VO_2 max (n=586), and apolipoprotein E (APOE) ε4 status (n=553)

		Model 1: 400m walk time			Model 2: VO_2 max			Model 3: APOE ε4		
		β	SE	*p*-value	β	SE	*p*-value	β	SE	*p*-value
**Global**	Brain aging score (SPARE-BA)	-0.0046	0.0020	**0.0220**	-0.0042	0.0021	0.0522	-0.0056	0.0021	**0.0078**
	AD score (SPARE-AD)	-0.0051	0.0034	0.1378	-0.0068	0.0036	0.0593	-0.0048	0.0036	0.1903
**Ventricles**	Ventricle	-0.0633	0.0370	0.0873	-0.0733	0.0390	0.0603	-0.0742	0.0414	0.0730
	Lateral ventricle	-0.0551	0.0338	0.1035	-0.0645	0.0357	0.0711	-0.0629	0.0379	0.0969
	3rd ventricle	-0.0022	0.0011	0.0528	-0.0019	0.0012	0.1091	-0.0025	0.0012	**0.0442**
	4th ventricle	-0.0025	0.0010	**0.0148**	-0.0019	0.0011	0.0676	-0.0026	0.0010	**0.0139**
**Frontal**	Frontal pole	0.0011	0.0023	0.6307	0.0006	0.0024	0.7988	0.0012	0.0023	0.6224
	Superior frontal gyrus	0.0077	0.0073	0.2912	0.0058	0.0078	0.4586	0.0092	0.0075	0.2172
	Middle frontal gyrus	0.0119	0.0082	0.1484	0.0073	0.0086	0.3979	0.0095	0.0087	0.2744
	Orbital part of inferior frontal gyrus	0.0014	0.0011	0.2379	0.0016	0.0012	0.1826	0.0011	0.0013	0.3900
	Triangular part of inferior frontal gyrus	0.0010	0.0020	0.6010	0.0006	0.0021	0.7835	0.0016	0.0021	0.4323
	Opercular part of inferior frontal gyrus	0.0039	0.0019	**0.0431**	0.0034	0.0020	0.0920	0.0044	0.0020	**0.0268**
	Medial frontal cortex	0.0010	0.0012	0.4361	0.0020	0.0013	0.1181	0.0019	0.0013	0.1251
	Anterior orbital gyrus	0.0010	0.0013	0.4233	0.0002	0.0013	0.8868	0.0012	0.0014	0.3863
	Posterior orbital gyrus	0.0040	0.0015	**0.0090**	0.0035	0.0016	**0.0285**	0.0048	0.0016	**0.0020**
	Lateral orbital gyrus	0.0012	0.0014	0.3807	0.0009	0.0015	0.5500	0.0013	0.0014	0.3704
	Medial orbital gyrus	0.0035	0.0021	0.1057	0.0027	0.0023	0.2339	0.0048	0.0023	**0.0345**
	Supplementary motor area	0.0022	0.0036	0.5458	0.0003	0.0038	0.9309	0.0019	0.0038	0.6073
	Subcallosal area	0.0028	0.0011	**0.0086**	0.0025	0.0011	**0.0242**	0.0030	0.0011	**0.0063**
	Insula	0.0074	0.0034	**0.0319**	0.0088	0.0037	**0.0165**	0.0077	0.0036	**0.0333**
	Operculum	0.0078	0.0039	**0.0447**	0.0074	0.0041	0.0695	0.0102	0.0041	**0.0129**
	Precentral gyrus	0.0140	0.0056	**0.0131**	0.0113	0.0060	0.0603	0.0157	0.0059	**0.0076**
	Frontal white matter	0.0559	0.0313	0.0749	0.0419	0.0320	0.1911	0.0475	0.0331	0.1517
**Parietal**	Postcentral gyrus	0.0088	0.0045	**0.0496**	0.0064	0.0048	0.1849	0.0095	0.0047	**0.0444**
	Superior parietal lobe	0.0056	0.0048	0.2447	0.0028	0.0052	0.5913	0.0054	0.0050	0.2772
	Precuneus	0.0087	0.0053	0.1049	0.0071	0.0058	0.2227	0.0098	0.0056	0.0800
	Supramarginal gyrus	0.0074	0.0039	0.0592	0.0052	0.0042	0.2218	0.0056	0.0042	0.1801
	Angular gyrus	0.0048	0.0051	0.3467	0.0026	0.0054	0.6263	0.0060	0.0053	0.2551
	Entorhinal cortex	0.0051	0.0019	**0.0073**	0.0054	0.0020	**0.0083**	0.0058	0.0020	**0.0037**
	Parahippocampal gyrus	0.0055	0.0021	**0.0074**	0.0051	0.0022	**0.0195**	0.0057	0.0022	**0.0086**
	Parietal white matter	0.0172	0.0169	0.3112	0.0136	0.0171	0.4274	0.0120	0.0178	0.5007
**Temporal**	Temporal pole	0.0106	0.0052	**0.0420**	0.0097	0.0055	0.0794	0.0105	0.0054	0.0530
	Superior temporal gyrus	0.0050	0.0035	0.1505	0.0044	0.0037	0.2304	0.0063	0.0037	0.0861
	Middle temporal gyrus	0.0164	0.0073	**0.0250**	0.0143	0.0077	0.0640	0.0187	0.0076	**0.0138**
	Inferior temporal gyrus	0.0097	0.0062	0.1191	0.0091	0.0065	0.1630	0.0123	0.0064	0.0547
	Fusiform gyrus	0.0028	0.0039	0.4744	0.0015	0.0041	0.7119	0.0027	0.0041	0.5086
	Temporal white matter	0.0390	0.0149	**0.0088**	0.0358	0.0158	**0.0231**	0.0266	0.0160	0.0967
**Occipital**	Occipital pole	0.0016	0.0024	0.5129	0.0011	0.0026	0.6732	0.0023	0.0025	0.3643
	Superior occipital gyrus	0.0058	0.0018	**0.0017**	0.0058	0.0019	**0.0029**	0.0055	0.0019	**0.0040**
	Middle occipital gyrus	0.0080	0.0033	**0.0140**	0.0059	0.0035	0.0931	0.0084	0.0034	**0.0144**
	Inferior occipital gyrus	0.0063	0.0037	0.0903	0.0068	0.0039	0.0856	0.0078	0.0039	**0.0437**
	Occipital fusiform gyrus	0.0069	0.0030	**0.0233**	0.0064	0.0031	**0.0418**	0.0072	0.0032	**0.0253**
	Medial occipital	0.0149	0.0078	0.0559	0.0124	0.0082	0.1310	0.0195	0.0081	**0.0154**
	Occipital white matter	-0.0027	0.0075	0.7162	-0.0025	0.0078	0.7487	-0.0062	0.0078	0.4279
**Cingulate**	Anterior cingulate gyrus	0.0065	0.0023	**0.0048**	0.0064	0.0025	**0.0104**	0.0079	0.0024	**0.0012**
	Middle cingulate gyrus	0.0046	0.0021	**0.0285**	0.0035	0.0022	0.1143	0.0062	0.0022	**0.0058**
	Posterior cingulate gyrus	0.0026	0.0019	0.1683	0.0024	0.0020	0.2322	0.0035	0.0020	0.0885
**Basal ganglia and related**	Accumbens area	0.0000	0.0004	0.9131	-0.0004	0.0004	0.3603	-0.0002	0.0004	0.6863
	Basal forebrain	0.0021	0.0010	**0.0260**	0.0024	0.0010	**0.0180**	0.0026	0.0010	**0.0095**
	Amygdala	0.0018	0.0008	**0.0258**	0.0019	0.0008	**0.0228**	0.0017	0.0008	**0.0447**
	Hippocampus	0.0019	0.0019	0.3254	0.0013	0.0020	0.5260	0.0014	0.0020	0.5017
	Caudate	0.0003	0.0022	0.8991	-0.0016	0.0023	0.4829	-0.0005	0.0022	0.8174
	Putamen	0.0039	0.0023	0.0996	0.0016	0.0025	0.5093	0.0042	0.0024	0.0894
	Globus pallidus	0.0015	0.0007	**0.0366**	0.0012	0.0008	0.1190	0.0009	0.0007	0.2014
	Thalamus	0.0048	0.0025	0.0514	0.0032	0.0026	0.2184	0.0047	0.0026	0.0725
	Anterior limb of the internal capsule	0.0003	0.0013	0.8054	-0.0011	0.0014	0.4472	0.0004	0.0014	0.7966
	Posterior limb of the internal capsule	0.0018	0.0013	0.1680	0.0005	0.0014	0.7083	0.0027	0.0014	0.0563
	Fornix	0.0007	0.0005	0.1987	0.0008	0.0005	0.1343	0.0009	0.0006	0.1091
**CC**	Corpus callosum	0.0023	0.0022	0.3065	0.0010	0.0023	0.6643	0.0013	0.0022	0.5709
**Cerebellum**	Cerebellum exterior gray matter	0.0077	0.0170	0.6495	0.0008	0.0180	0.9644	0.0094	0.0176	0.5940
	Cerebellum white matter	0.0094	0.0041	**0.0222**	0.0083	0.0045	0.0647	0.0081	0.0043	0.0579

Note: Statistical results are from linear mixed effects models with significance at two-sided $p<0.05$. Bold number indicates $p<0.05$. k_{PCr} and SPARE scores are standardized to Z scores. Magnetic resonance imaging (MRI) volumes are on the original scale in cm^3 for interpretation purposes. CC: Corpus callosum.

Supplementary Table 2. Associations between skeletal muscle oxidative capacity and white matter microstructural measures of axial diffusivity and radial diffusivity (n=639)

		Axial Diffusivity (lower value: higher integrity)					
		Cross-sectional associations			Longitudinal associations		
		β	SE	*p*-value	β	SE	*p*-value
**Commissural fibers**	Genu of corpus callosum	-0.0559	0.0342	0.1025	-0.0002	0.0050	0.9660
	Body of corpus callosum	-0.0402	0.0323	0.2141	-0.0028	0.0050	0.5710
	Splenium of corpus callosum	-0.0679	0.0364	0.0627	-0.0040	0.0041	0.3313
**Association fibers**	External capsule	-0.0017	0.0321	0.9571	-0.0003	0.0046	0.9534
	Uncinate fasciculus	-0.0003	0.0352	0.9929	0.0052	0.0047	0.2674
	Superior longitudinal fasciculus	-0.0010	0.0359	0.9777	0.0018	0.0055	0.7462
	Inferior longitudinal fasciculus	-0.0187	0.0400	0.6407	-0.0014	0.0072	0.8423
	Superior fronto-occipital fasciculus	-0.0040	0.0349	0.9076	0.0009	0.0058	0.8728
	Inferior fronto-occipital fasciculus	-0.0198	0.0333	0.5516	0.0001	0.0043	0.9802
	Cingulate part of the cingulum	-0.0446	0.0411	0.2789	0.0031	0.0067	0.6408
	Hippocampal part of the cingulum	-0.0787	0.0400	**0.0498**	0.0033	0.0073	0.6447
	Fornix (column and body)	-0.0388	0.0328	0.2378	0.0040	0.0035	0.2555
	Fornix (cres) Stria terminalis	-0.0449	0.0350	0.1997	0.0015	0.0049	0.7655
**Projection fibers**	Anterior limb of the internal capsule	-0.0341	0.0343	0.3196	0.0034	0.0055	0.5424
	Posterior limb of the internal capsule	-0.0492	0.0357	0.1688	-0.0051	0.0043	0.2409
	Anterior corona radiata	-0.0026	0.0392	0.9479	-0.0015	0.0053	0.7736
	Superior corona radiata	-0.0373	0.0359	0.2987	-0.0021	0.0041	0.6118
	Posterior corona radiata	-0.0333	0.0371	0.3691	-0.0046	0.0043	0.2866
**Cerebellum**	Cerebellar peduncle	-0.0411	0.0364	0.2595	-0.0022	0.0065	0.7364
		**Radial Diffusivity (lower value: higher integrity)**					
		**Cross-sectional associations**			**Longitudinal associations**		
		**β**	**SE**	***p*-value**	**β**	**SE**	***p*-value**
**Commissural fibers**	Genu of corpus callosum	-0.0552	0.0335	0.0995	-0.0025	0.0045	0.5847
	Body of corpus callosum	-0.0391	0.0346	0.2589	-0.0061	0.0038	0.1048
	Splenium of corpus callosum	-0.0872	0.0394	**0.0274**	-0.0054	0.0035	0.1237
**Association fibers**	External capsule	-0.0033	0.0291	0.9098	-0.0018	0.0037	0.6278
	Uncinate fasciculus	0.0041	0.0360	0.9089	0.0055	0.0046	0.2326
	Superior longitudinal fasciculus	0.0119	0.0350	0.7330	-0.0057	0.0038	0.1359
	Inferior longitudinal fasciculus	-0.0213	0.0383	0.5782	-0.0040	0.0043	0.3591
	Superior fronto-occipital fasciculus	-0.0161	0.0338	0.6339	-0.0018	0.0047	0.6980
	Inferior fronto-occipital fasciculus	-0.0110	0.0341	0.7468	-0.0058	0.0043	0.1807
	Cingulate part of the cingulum	-0.0539	0.0360	0.1352	-0.0065	0.0044	0.1377
	Hippocampal part of the cingulum	-0.0738	0.0377	0.0509	-0.0114	0.0054	**0.0360**
	Fornix (column and body)	-0.0432	0.0336	0.1985	0.0001	0.0026	0.9785
	Fornix (cres) Stria terminalis	-0.0549	0.0335	0.1012	-0.0027	0.0034	0.4393
**Projection fibers**	Anterior limb of the internal capsule	-0.0117	0.0331	0.7234	-0.0016	0.0044	0.7163
	Posterior limb of the internal capsule	0.0032	0.0364	0.9293	-0.0097	0.0061	0.1115
	Anterior corona radiata	0.0054	0.0321	0.8668	0.0006	0.0034	0.8590
	Superior corona radiata	0.0036	0.0345	0.9174	-0.0025	0.0039	0.5189
	Posterior corona radiata	0.0107	0.0387	0.7824	-0.0037	0.0041	0.3705
**Cerebellum**	Cerebellar peduncle	-0.0525	0.0371	0.1579	-0.0016	0.0073	0.8299

Note: Statistical results are derived from linear mixed effects models with significance at two-sided $p<0.05$. Bold number indicates $p<0.05$. k_{PCr} and diffusion tensor imaging (DTI) values are standardized to Z scores.

Supplementary Table 3. Longitudinal associations of skeletal muscle oxidative capacity with white matter microstructural changes of fractional anisotropy and mean diffusivity after adjusting for 400m time (n=617), VO_2 max (n=578), and apolipoprotein E (APOE) ε4 carrier status (n=545)

		Fractional Anisotropy (higher value: higher integrity)								
		Model 1: 400m walk time			Model 2: VO_2 max			Model 3: APOE ε4		
		β	SE	*p*-value	β	SE	*p*-value	β	SE	*p*-value
**Commissural fibers**	Genu of corpus callosum	0.0015	0.0045	0.7369	-0.0003	0.0047	0.9477	0.0009	0.0046	0.8453
	Body of corpus callosum	0.0062	0.0036	0.0892	0.0062	0.0038	0.1035	0.0073	0.0037	**0.0490**
	Splenium of corpus callosum	0.0061	0.0038	0.1073	0.0063	0.0040	0.1173	0.0093	0.0040	0.0208
**Association fibers**	External capsule	0.0014	0.0050	0.7876	-0.0008	0.0052	0.8743	0.0029	0.0053	0.5845
	Uncinate fasciculus	-0.0036	0.0048	0.4553	-0.0039	0.0050	0.4434	-0.0033	0.0050	0.5160
	Superior longitudinal fasciculus	0.0079	0.0037	**0.0332**	0.0055	0.0039	0.1601	0.0083	0.0039	**0.0319**
	Inferior longitudinal fasciculus	0.0031	0.0042	0.4564	0.0017	0.0045	0.7004	0.0033	0.0043	0.4456
	Superior fronto-occipital fasciculus	0.0060	0.0052	0.2509	0.0041	0.0052	0.4314	0.0096	0.0053	0.0700
	Inferior fronto-occipital fasciculus	0.0067	0.0054	0.2203	0.0054	0.0056	0.3313	0.0055	0.0056	0.3306
	Cingulate part of the cingulum	0.0051	0.0037	0.1726	0.0029	0.0037	0.4278	0.0079	0.0037	**0.0347**
	Hippocampal part of the cingulum	0.0120	0.0058	**0.0383**	0.0092	0.0058	0.1121	0.0108	0.0063	0.0869
	Fornix (column and body)	0.0021	0.0029	0.4615	0.0007	0.0031	0.8240	0.0007	0.0031	0.8125
	Fornix (cres) Stria terminalis	0.0074	0.0040	0.0663	0.0082	0.0043	0.0555	0.0085	0.0042	**0.0411**
**Projection fibers**	Anterior limb of the internal capsule	0.0038	0.0050	0.4511	0.0018	0.0052	0.7212	0.0058	0.0051	0.2606
	Posterior limb of the internal capsule	0.0082	0.0063	0.1928	0.0036	0.0065	0.5771	0.0080	0.0063	0.2032
	Anterior corona radiata	-0.0005	0.0030	0.8793	-0.0007	0.0031	0.8165	0.0005	0.0032	0.8830
	Superior corona radiata	0.0017	0.0036	0.6464	-0.0002	0.0039	0.9646	0.0019	0.0037	0.6073
	Posterior corona radiata	0.0035	0.0039	0.3717	0.0025	0.0043	0.5589	0.0038	0.0040	0.3441
**Cerebellum**	Cerebellar peduncle	0.0009	0.0081	0.9066	-0.0031	0.0084	0.7124	0.0029	0.0084	0.7312

		Mean Diffusivity (lower value: higher integrity)								
		Model 1: 400m walk time			Model 2: VO_2 max			Model 3: APOE ε4		
		β	SE	*p*-value	β	SE	*p*-value	β	SE	*p*-value
**Commissural fibers**	Genu of corpus callosum	-0.0032	0.0047	0.4992	-0.0003	0.0047	0.9448	-0.0008	0.0047	0.8670
	Body of corpus callosum	-0.0054	0.0040	0.1789	-0.0058	0.0043	0.1750	-0.0039	0.0041	0.3499
	Splenium of corpus callosum	-0.0039	0.0039	0.3204	-0.0045	0.0041	0.2729	-0.0064	0.0040	0.1076
**Association fibers**	External capsule	-0.0008	0.0036	0.8167	0.0017	0.0038	0.6543	-0.0028	0.0037	0.4551
	Uncinate fasciculus	0.0070	0.0048	0.1450	0.0062	0.0049	0.2074	0.0063	0.0047	0.1878
	Superior longitudinal fasciculus	-0.0043	0.0043	0.3228	-0.0023	0.0047	0.6205	-0.0057	0.0045	0.2072
	Inferior longitudinal fasciculus	-0.0044	0.0059	0.4607	-0.0038	0.0065	0.5510	-0.0060	0.0059	0.3047
	Superior fronto-occipital fasciculus	-0.0010	0.0050	0.8376	-0.0016	0.0051	0.7616	-0.0032	0.0051	0.5252
	Inferior fronto-occipital fasciculus	-0.0053	0.0041	0.1926	-0.0037	0.0043	0.3892	-0.0047	0.0042	0.2579
	Cingulate part of the cingulum	-0.0038	0.0055	0.4915	0.0000	0.0056	0.9986	-0.0050	0.0056	0.3693
	Hippocampal part of the cingulum	-0.0046	0.0062	0.4595	-0.0051	0.0061	0.4064	-0.0085	0.0060	0.1594
	Fornix (column and body)	0.0014	0.0027	0.5960	0.0031	0.0028	0.2599	0.0024	0.0028	0.3987
	Fornix (cres) Stria terminalis	0.0000	0.0038	0.9999	0.0003	0.0039	0.9473	-0.0017	0.0039	0.6627
**Projection fibers**	Anterior limb of the internal capsule	-0.0010	0.0048	0.8388	0.0003	0.0050	0.9477	-0.0018	0.0047	0.7105
	Posterior limb of the internal capsule	-0.0120	0.0057	**0.0338**	-0.0091	0.0059	0.1196	-0.0114	0.0057	**0.0440**
	Anterior corona radiata	0.0003	0.0039	0.9365	-0.0012	0.0038	0.7584	-0.0016	0.0039	0.6764
	Superior corona radiata	-0.0023	0.0040	0.5734	-0.0017	0.0043	0.6982	-0.0030	0.0041	0.4712
	Posterior corona radiata	-0.0036	0.0038	0.3347	-0.0056	0.0045	0.2159	-0.0064	0.0044	0.1462
**Cerebellum**	Cerebellar peduncle	-0.0005	0.0072	0.9429	0.0023	0.0074	0.7589	0.0010	0.0073	0.8903

Note: Statistical results are derived from linear mixed effects models with significance at two-sided $p<0.05$. Bold number indicates $p<0.05$. k_{PCr} and diffusion tensor imaging (DTI) values are standardized to Z scores.

Regional brain volumetric measures via MRI with adjustment for total brain volume

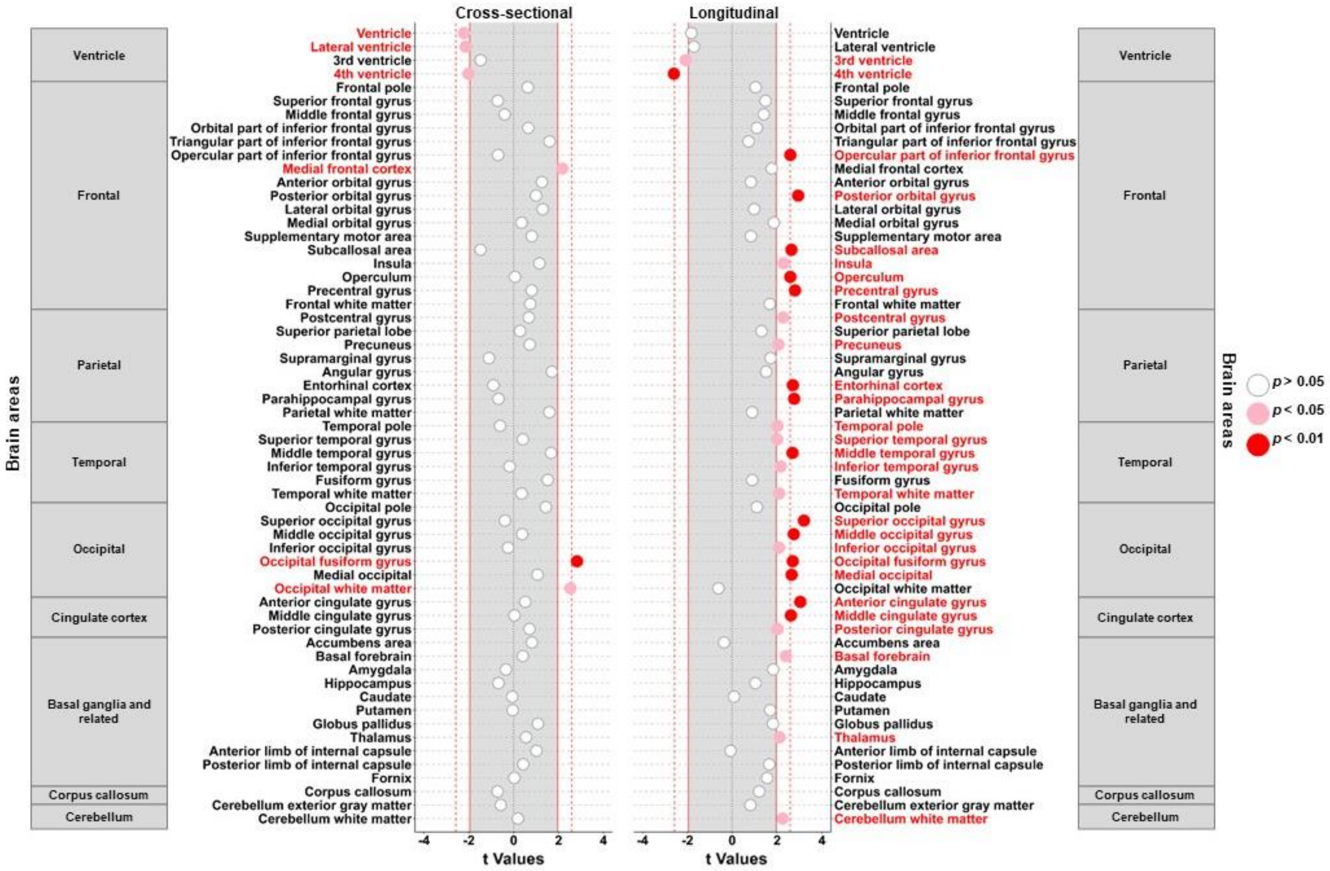

Supplementary Figure 1. Dot plots for associations between skeletal muscle oxidative capacity and brain volumetric measures via magnetic resonance imaging (MRI) with adjustment for total brain volume (n=649). Legend: Statistical results are derived from linear mixed effects models with significance at two-sided $p<0.05$. Data points beyond red solid lines are at $p<0.05$ and beyond red dotted lines are at $p<0.01$. k_{PCr} is standardized to Z scores. Magnetic resonance imaging (MRI) volumes are on the original scale in cm^3 for interpretation purposes.

Regional brain volumetric measures via MRI after exclusion of data points at and after cognitive impairment

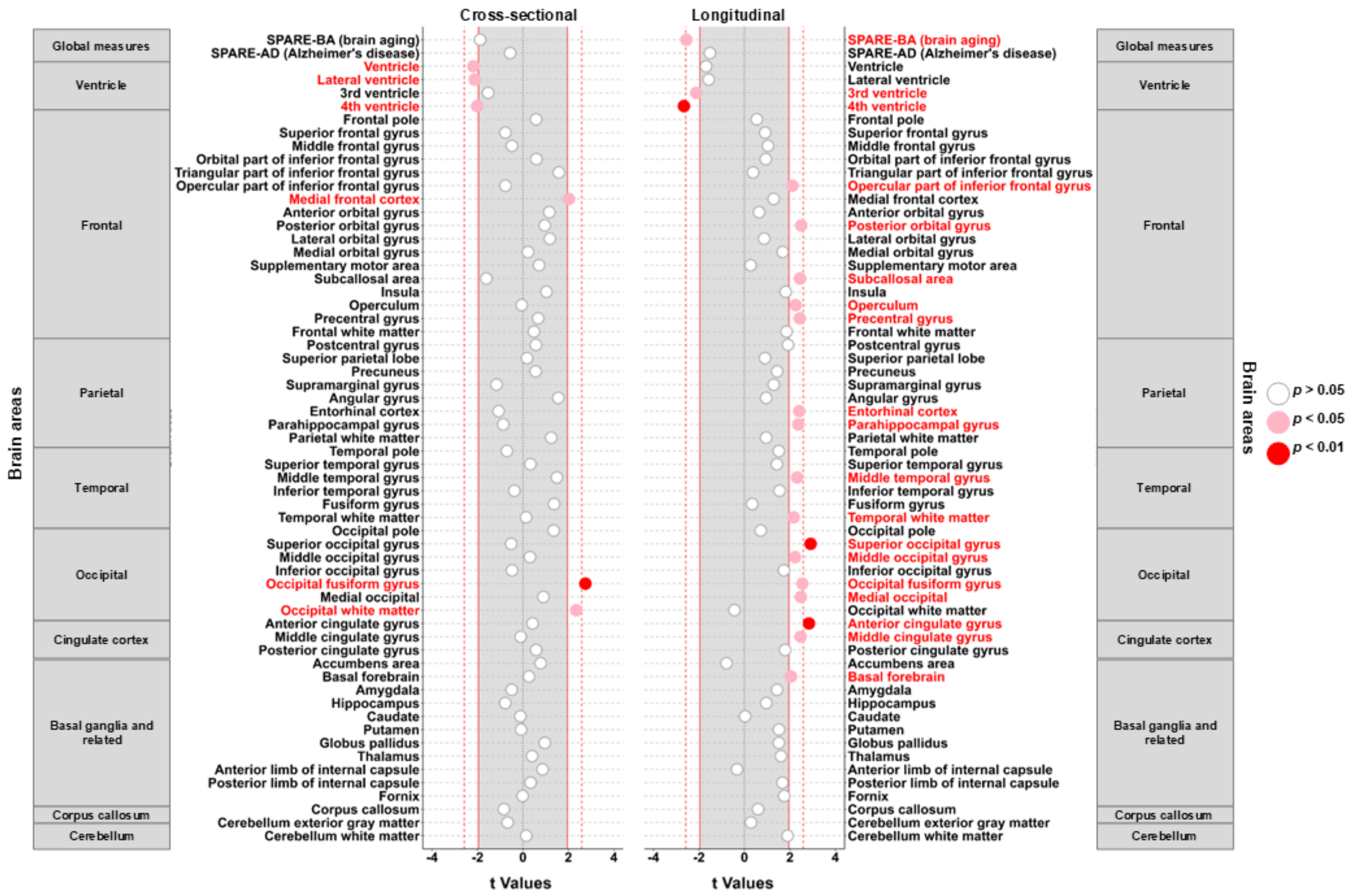

Supplementary Figure 2. Dot plots for associations between skeletal muscle oxidative capacity and brain volumetric measures via magnetic resonance imaging (MRI) after exclusion of data points at and after cognitive impairment (n=643). Legend: Statistical results are derived from linear mixed effects models with significance at two-sided $p<0.05$. Data points beyond red solid lines are at $p<0.05$ and beyond red dotted lines are at $p<0.01$. k_{PCr} is standardized to Z scores. Magnetic resonance imaging (MRI) volumes are on the original scale in cm^3 for interpretation purposes.

Regional brain volumetric measures via MRI for participants aged 60 and above

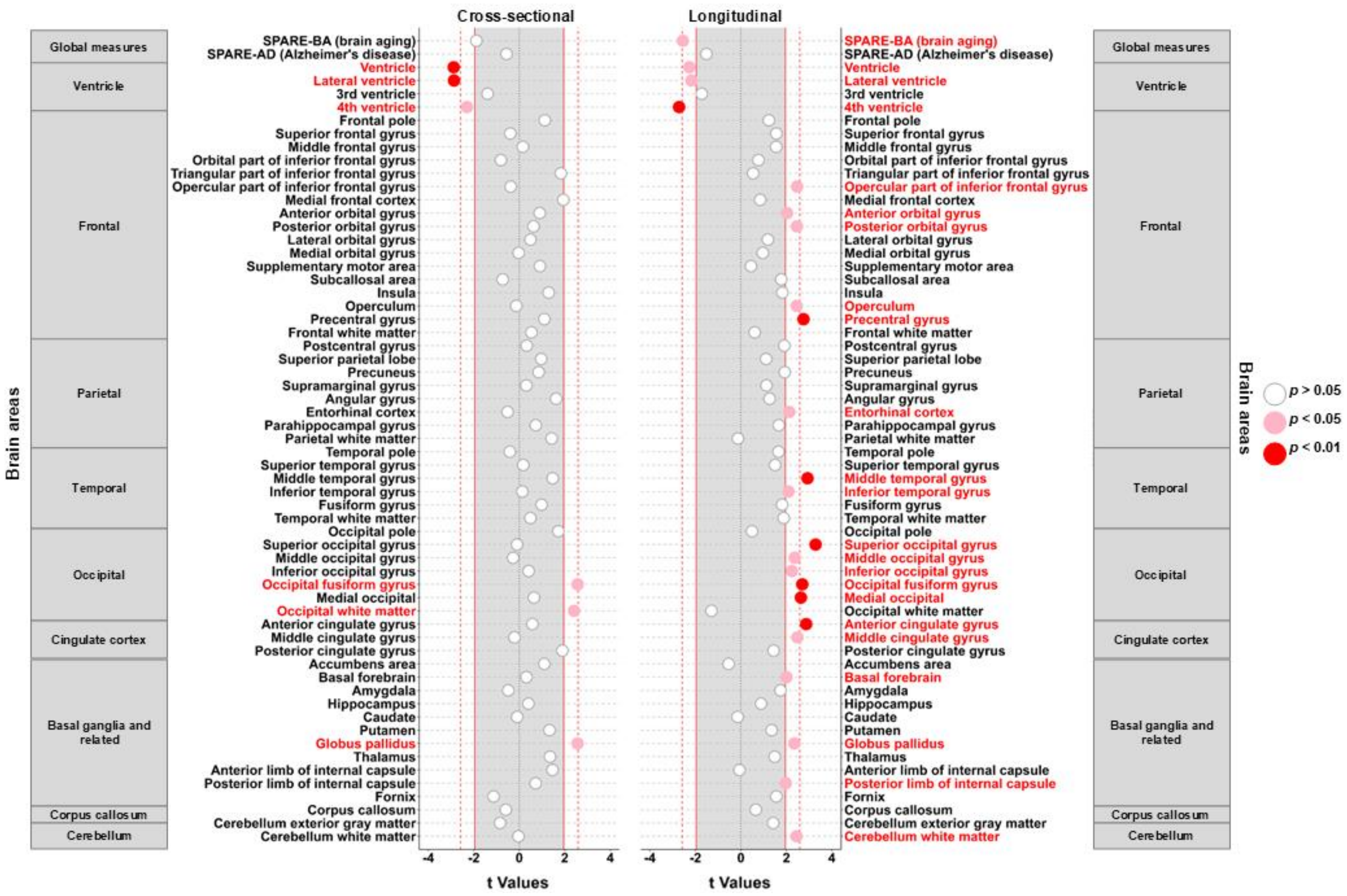

Supplementary Figure 3. Dot plots for associations between skeletal muscle oxidative capacity and brain volumetric measures via magnetic resonance imaging (MRI) after restricting to participants aged 60 and above (n=451). Legend: Statistical results are derived from linear mixed effects models with significance at two-sided $p<0.05$. Data points beyond red solid lines are at $p<0.05$ and beyond red dotted lines are at $p<0.01$. k_{PCr} is standardized to Z scores. Magnetic resonance imaging (MRI) volumes are on the original scale in cm^3 for interpretation purposes.

Gray matter microstructure via DTI

a

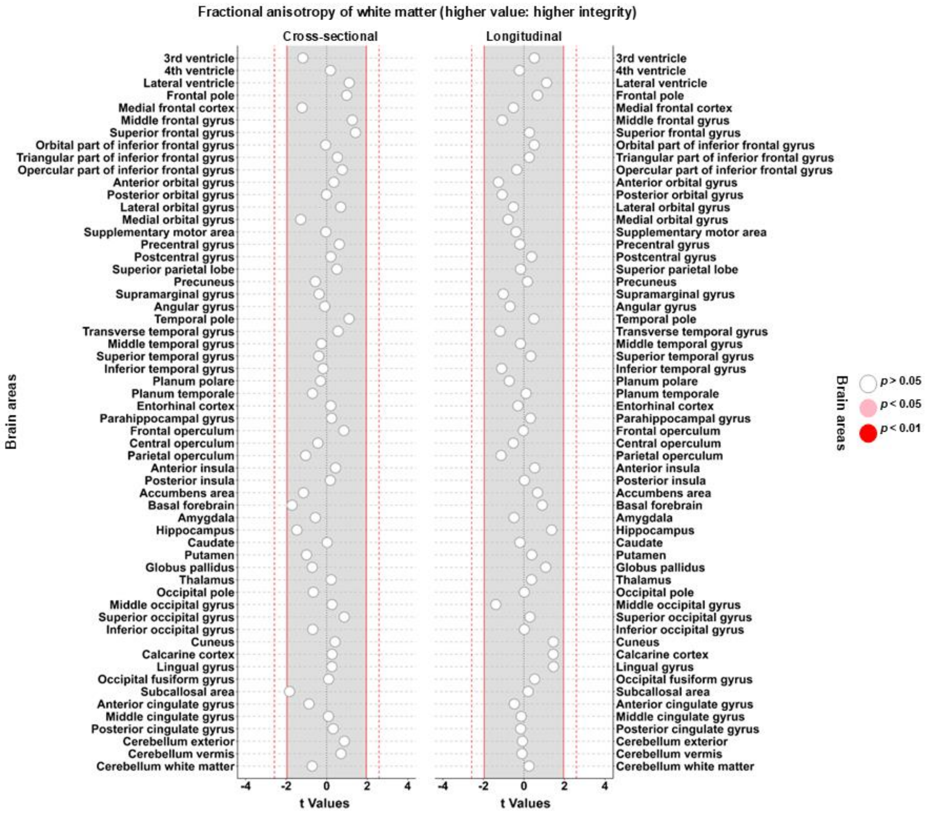

b

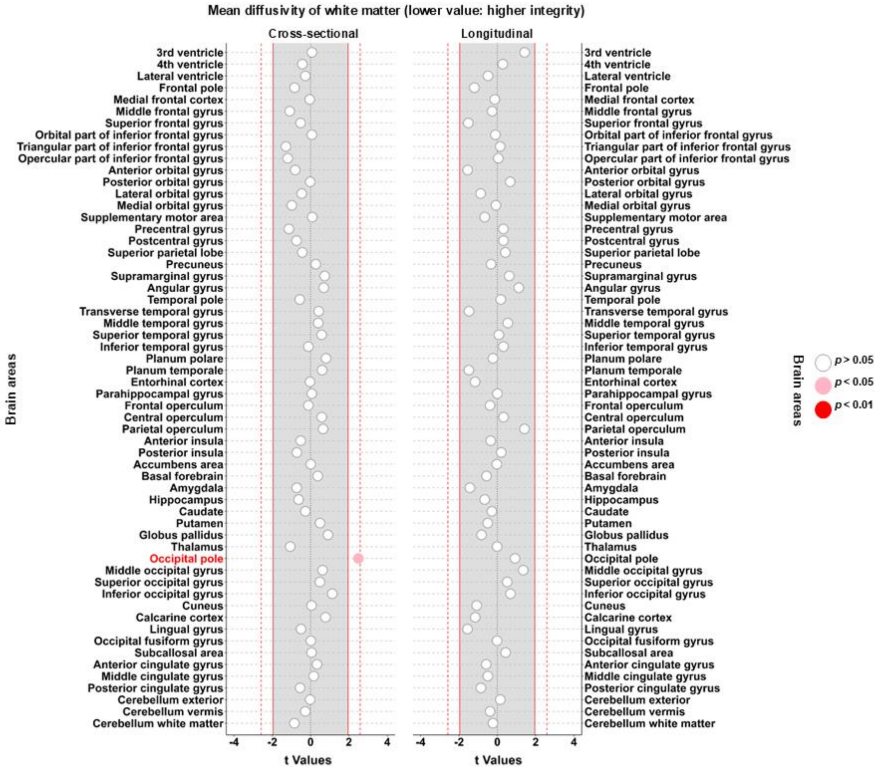

Supplementary Figure 4. Dot plots for associations between skeletal muscle oxidative capacity and gray matter diffusion tensor imaging (DTI) measures of fractional anisotropy (FA) (a) and mean diffusivity (MD) (b) (n=632). Legend: Statistical results are derived from linear mixed effects models with significance at two-sided p<0.05. Data points beyond red solid lines are at p<0.05 and beyond red dotted lines are at p<0.01. k_{PCr} and diffusion tensor imaging (DTI) values are standardized to Z scores.

White matter microstructure via DTI after exclusion of data points at and after cognitive impairment

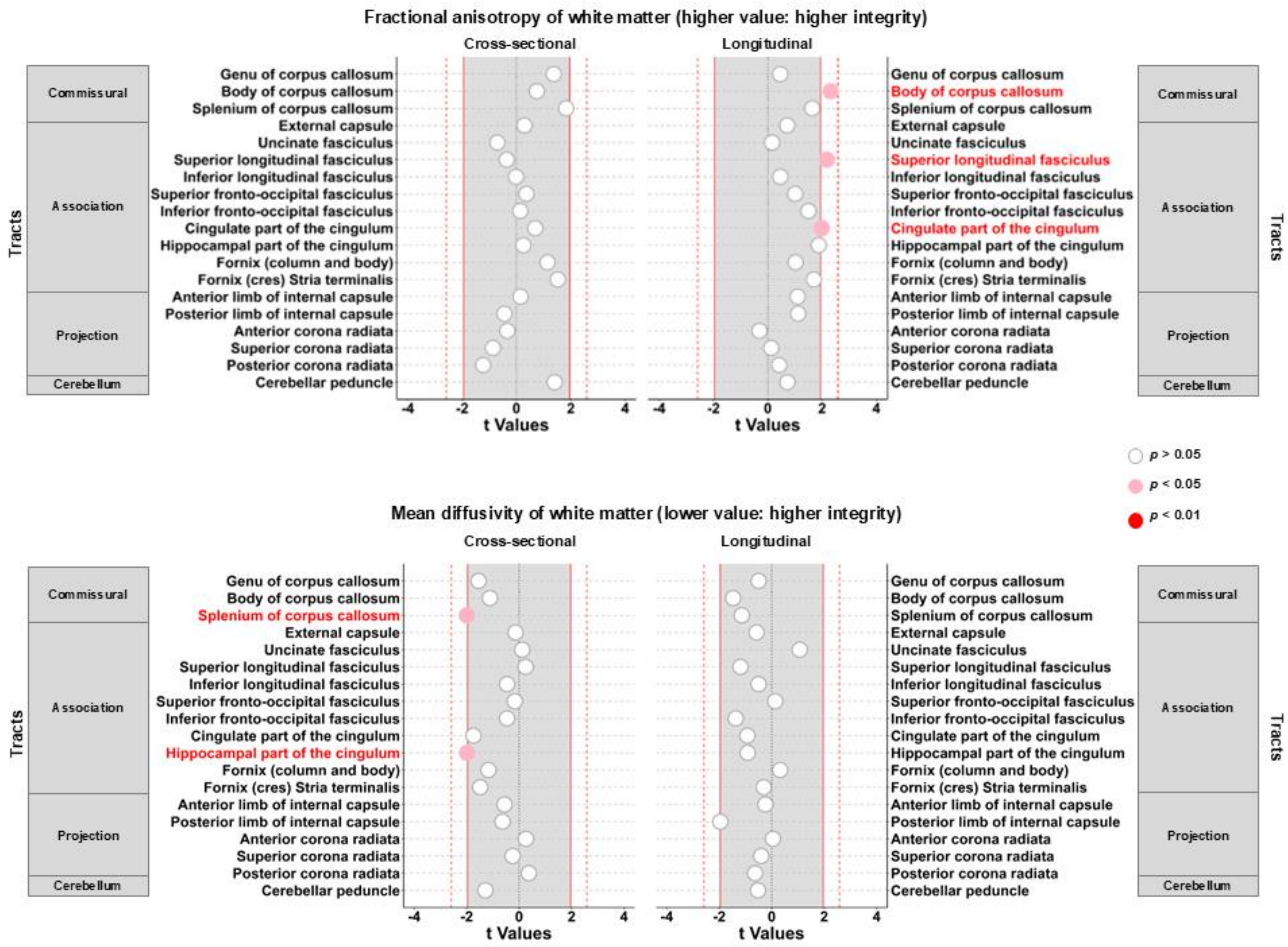

Supplementary Figure 5. Dot plots for associations between skeletal muscle oxidative capacity and white matter diffusion tensor imaging (DTI) measures of fractional anisotropy (FA) and mean diffusivity (MD) after exclusion of data points at and after cognitive impairment (n=632). Legend: Statistical results are derived from a linear mixed effects models with significance at two-sided $p<0.05$. Data points beyond red solid lines are at $p<0.05$ and beyond red dotted lines are at $p<0.01$. k_{PCr} and diffusion tensor imaging (DTI) values are standardized to Z scores.

White matter microstructure via DTI for participants aged 60 and above

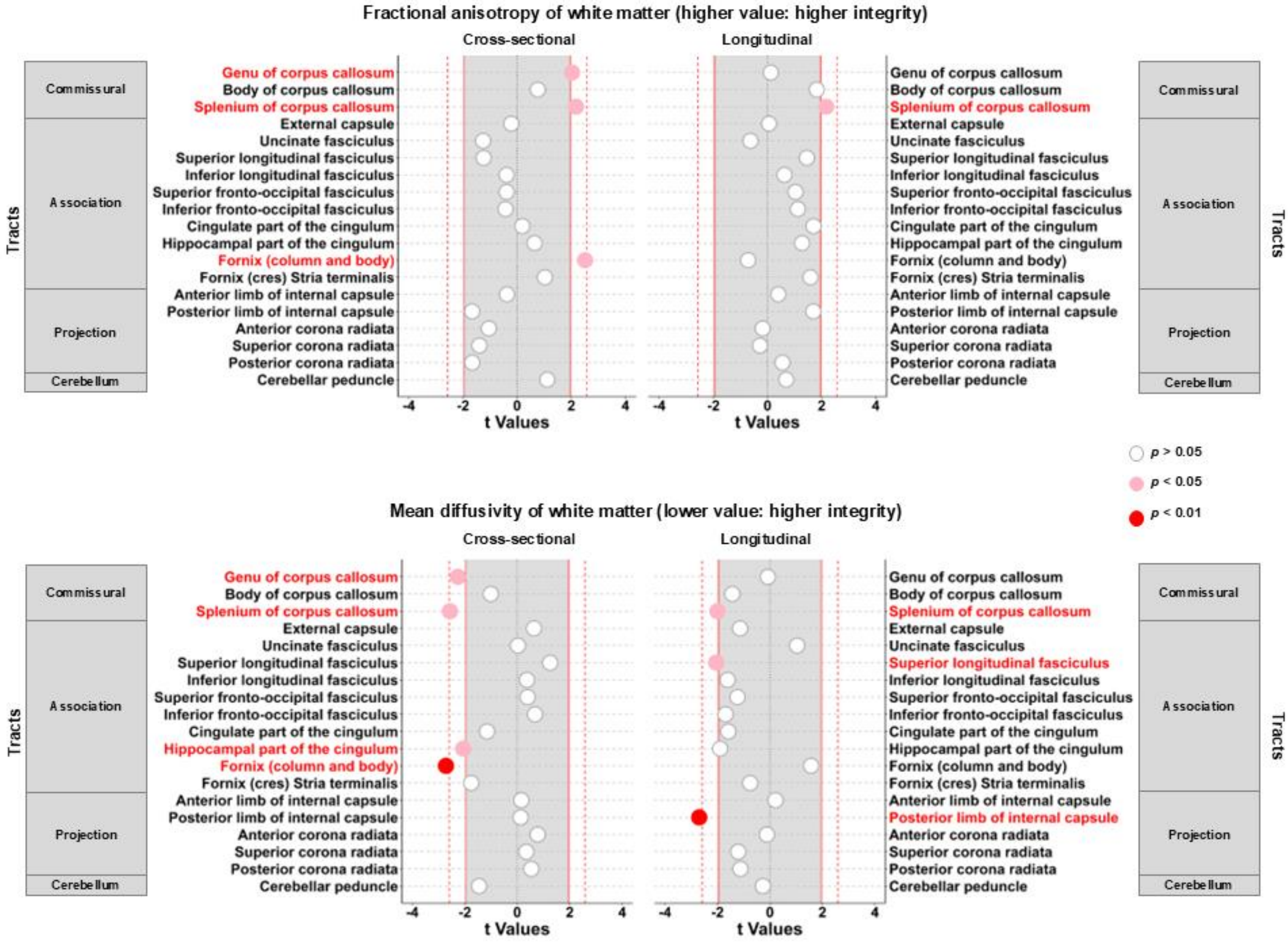

Supplementary Figure 6. Dot plots for associations between skeletal muscle oxidative capacity and white matter diffusion tensor imaging (DTI) measures of fractional anisotropy (FA) and mean diffusivity (MD) after restricting to participants aged 60 and above (n=444). Legend: Statistical results are derived from a linear mixed effects models with significance at two-sided $p<0.05$. Data points beyond red solid lines are at $p<0.05$ and beyond red dotted lines are at $p<0.01$. k_{PCr} and diffusion tensor imaging (DTI) values are standardized to Z scores.

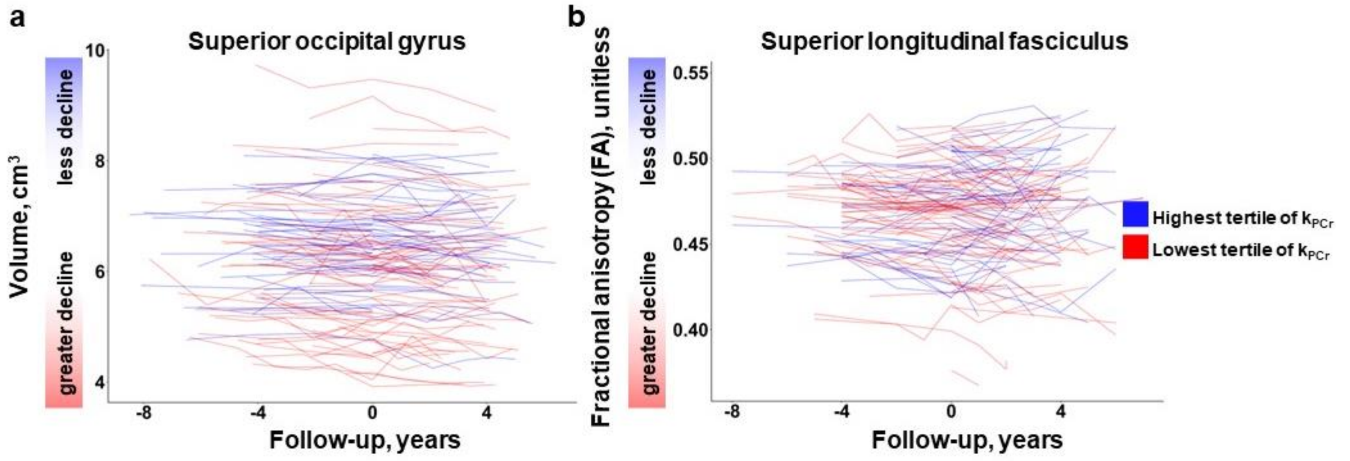

Supplementary Figure 7. Individual data points of volume of superior occipital gyrus (n=649) (a) and fractional anisotropy of the superior longitudinal fasciculus (n=639) (b) in those with lowest and highest tertiles of skeletal muscle oxidative capacity. Legend: A 50% random sample was shown in this plot for better visualization.
