# Supplementary material for: Higher skeletal muscle mitochondrial oxidative capacity is associated with preserved brain structure up to over a decade
Source: Nat Commun. 2024 Dec 30;15:10786. doi: 10.1038/s41467-024-55009-z (PMC11686348; doi:10.1038/s41467-024-55009-z)
Supplement: Supplementary file 2 — Description of Additional Supplementary Files [file 41467_2024_55009_MOESM2_ESM.pdf]

## Description of Additional Supplementary Files:

**Supplementary Dataset 1:** The script used for the linear mixed effects modeling on the associations between muscle oxidative capacity and longitudinal changes in brain MRI and DTI outcomes.
