# Supplementary material for: Higher skeletal muscle mitochondrial oxidative capacity is associated with preserved brain structure up to over a decade
Source: Nat Commun. 2024 Dec 30;15:10786. doi: 10.1038/s41467-024-55009-z (PMC11686348; doi:10.1038/s41467-024-55009-z)
Supplement: Supplementary file 4 — Reporting Summary [file 41467_2024_55009_MOESM4_ESM.pdf]

nature portfolio

Corresponding author(s): Qu Tian, PhD
Last updated by author(s): Nov 1, 2024

Reporting Summary

Nature Portfolio wishes to improve the reproducibility of the work that we publish. This form provides structure for consistency and transparency in reporting. For further information on Nature Portfolio policies, see our Editorial Policies and the Editorial Policy Checklist.

Statistics

For all statistical analyses, confirm that the following items are present in the figure legend, table legend, main text, or Methods section.

n/a	Confirmed	
☐	☒	The exact sample size (*n*) for each experimental group/condition, given as a discrete number and unit of measurement
☐	☒	A statement on whether measurements were taken from distinct samples or whether the same sample was measured repeatedly
☐	☒	The statistical test(s) used AND whether they are one- or two-sided<br>*Only common tests should be described solely by name; describe more complex techniques in the Methods section.*
☐	☒	A description of all covariates tested
☐	☒	A description of any assumptions or corrections, such as tests of normality and adjustment for multiple comparisons
☐	☒	A full description of the statistical parameters including central tendency (e.g. means) or other basic estimates (e.g. regression coefficient) AND variation (e.g. standard deviation) or associated estimates of uncertainty (e.g. confidence intervals)
☐	☒	For null hypothesis testing, the test statistic (e.g. *F*, *t*, *r*) with confidence intervals, effect sizes, degrees of freedom and *P* value noted<br>*Give P values as exact values whenever suitable.*
☒	☐	For Bayesian analysis, information on the choice of priors and Markov chain Monte Carlo settings
☒	☐	For hierarchical and complex designs, identification of the appropriate level for tests and full reporting of outcomes
☒	☐	Estimates of effect sizes (e.g. Cohen's *d*, Pearson's *r*), indicating how they were calculated

Our web collection on statistics for biologists contains articles on many of the points above.

Software and code

Policy information about availability of computer code

Data collection	No software was used.
Data analysis	The linear mixed effects model code is provided as supplementary data. Additional analytical codes are available upon request. All analyses were performed using the "nlme" package of Rstudio version 4.3.1 (Boston, MA).

For manuscripts utilizing custom algorithms or software that are central to the research but not yet described in published literature, software must be made available to editors and reviewers. We strongly encourage code deposition in a community repository (e.g. GitHub). See the Nature Portfolio guidelines for submitting code & software for further information.

Data

Policy information about availability of data

All manuscripts must include a data availability statement. This statement should provide the following information, where applicable:

- Accession codes, unique identifiers, or web links for publicly available datasets
- A description of any restrictions on data availability
- For clinical datasets or third party data, please ensure that the statement adheres to our policy

Data analyzed in this study are available upon request by proposal submission via the BLSA website portal (https://www.blsa.nih.gov/how-apply). All requests to access the BLSA datasets are reviewed by the BLSA Data Sharing Proposal Review Committee and are also subject to approval from the NIH Institutional Review Board.

Research involving human participants, their data, or biological material

Policy information about studies with human participants or human data. See also policy information about sex, gender (identity/presentation), and sexual orientation and race, ethnicity and racism.

Reporting on sex and gender	Details on information on sex was reported previously: "Information on sex (i.e., men, women) was collected by self-report from the participants using a questionnaire at an in-person interview during the BLSA visit. Participants answered this question orally and the answer was recorded by the staff." Reference: Tian, Q., Mitchell, B. A., Erus, G., Davatzikos, C., Moaddel, R., Resnick, S. M., & Ferrucci, L. (2023). Sex differences in plasma lipid profiles of accelerated brain aging. Neurobiology of Aging, 129, 178-184.
Reporting on race, ethnicity, or other socially relevant groupings	Information on race, ethnicity, or other socially relevant groupings were collected by self-report from the participants using a questionnaire at an in-person interview during the BLSA visit.
Population characteristics	Participants' characteristics are reported in Table 1, including demographic factors and lifestyle factors (i.e, physical activity).
Recruitment	Participants are volunteers from the community. Those who meet the eligibility at the enrollment are recruited in the study. Once enrolled, they are continuously followed up over time. Participants tend to be healthier than the general adult population due to their voluntary participation to the study and eligibility to the brain MRI.
Ethics oversight	The BLSA protocol was approved by the Institutional Review Board of the National Institutes of Health.

Note that full information on the approval of the study protocol must also be provided in the manuscript.

Field-specific reporting

Please select the one below that is the best fit for your research. If you are not sure, read the appropriate sections before making your selection.

- [x] Life sciences
- [ ] Behavioural & social sciences
- [ ] Ecological, evolutionary & environmental sciences

For a reference copy of the document with all sections, see nature.com/documents/nr-reporting-summary-flat.pdf

Life sciences study design

All studies must disclose on these points even when the disclosure is negative.

Sample size	Sample selection was reported in Figure 1 and Methods section 2.1, "In this study, we used the first assessment of skeletal muscle mitochondrial function to examine the relationship with longitudinal changes in brain structure between 2008 and 2020. A sample of 649 participants were included for analysis." All eligible participants were included in the analysis.
Data exclusions	Participants are a convenience sample from the BLSA, and all had measures of interest.
Replication	Several sensitivity analyses were performed to test the strength of the associations. Please see statistical analysis section last paragraph, "To understand whether global atrophy affected the association with regional brain atrophy, in a sensitivity analysis we adjusted for total brain volume at the first MRI visit instead of intracranial volume. To understand how cognitive impairment affected the longitudinal associations, we repeated the analyses by removing data points at and after symptom onset of cognitive impairment.". Results remain similar in these sensitivity analyses.
Randomization	NA
Blinding	NA

Reporting for specific materials, systems and methods

We require information from authors about some types of materials, experimental systems and methods used in many studies. Here, indicate whether each material, system or method listed is relevant to your study. If you are not sure if a list item applies to your research, read the appropriate section before selecting a response.

Materials & experimental systems

n/a	Involved in the study
☒	☐ Antibodies
☒	☐ Eukaryotic cell lines
☒	☐ Palaeontology and archaeology
☒	☐ Animals and other organisms
☒	☐ Clinical data
☒	☐ Dual use research of concern
☒	☐ Plants

Methods

n/a	Involved in the study
☒	☐ ChIP-seq
☒	☐ Flow cytometry
☐	☒ MRI-based neuroimaging

Plants

Seed stocks

Report on the source of all seed stocks or other plant material used. If applicable, state the seed stock centre and catalogue number. If plant specimens were collected from the field, describe the collection location, date and sampling procedures.

Novel plant genotypes

Describe the methods by which all novel plant genotypes were produced. This includes those generated by transgenic approaches, gene editing, chemical/radiation-based mutagenesis and hybridization. For transgenic lines, describe the transformation method, the number of independent lines analyzed and the generation upon which experiments were performed. For gene-edited lines, describe the editor used, the endogenous sequence targeted for editing, the targeting guide RNA sequence (if applicable) and how the editor was applied.

Authentication

Describe any authentication procedures for each seed stock used or novel genotype generated. Describe any experiments used to assess the effect of a mutation and, where applicable, how potential secondary effects (e.g. second site T-DNA insertions, mosiacism, off-target gene editing) were examined.

Magnetic resonance imaging

Experimental design

Design type: Structural neuroimaging data via MRI and DTI.

Design specifications: NA

Behavioral performance measures: NA

Acquisition

Imaging type(s): Structural neuroimaging data via MRI and DTI.

Field strength: 3T

Sequence & imaging parameters: Please see details in section 2.3 for brain MRI and 2.4 for brain DTI.

Area of acquisition: Imaging data were acquired on 3 comparable 3T Philips Achieva scanners at the Kennedy Krieger Institute (KKI) or the National Institute on Aging (NIA) in Baltimore, Maryland.

Diffusion MRI ☒ Used ☐ Not used

Parameters: Please see section 2.4.

Preprocessing

Preprocessing software: In brief, “multiple atlases with semiautomatically extracted ground-truth regions of interest (ROI) labels are first warped individually to the target image using a nonlinear registration method. The ensemble is fused into a final consensus segmentation. This workflow for segmenting the brain into a set of anatomical ROIs has been previously validated extensively in the BLSA MRI dataset (Erus et al., 2018). Notably, the MUSE anatomically labeling approach is robust and accurate, owing to its use of multiple atlases and multiple registration methods. This ensemble approach has consistently outperformed segmentations using individual warping methods alone and has achieved high accuracy in several benchmark datasets (Doshi et al., 2016). The MUSE methodology has been used for processing thousands of scans from various datasets, producing robust and consistent results. MUSE is available through the image processing portal: ipp.cbica.upenn.edu.”

Normalization: See above.

Normalization template: See above.

Noise and artifact removal: See above.

Volume censoring | See above.

Statistical modeling & inference

Model type and settings | Linear mixed effects model were used, including both random effect and fixed effects.

Effect(s) tested | Regression coefficients and confidence interval were reported.

Specify type of analysis: ☐ Whole brain ☐ ROI-based ☒ Both

Anatomical location(s) | Multiple regions and tracts of interest across the whole brain.

Statistic type for inference (See Eklund et al. 2016) | NA

Correction | In this exploratory analysis, we set significance at two-sided p<0.05 and we also reported FDR-adjusted p-values.

Models & analysis

n/a	Involved in the study
☒	☐ Functional and/or effective connectivity
☒	☐ Graph analysis
☐	☒ Multivariate modeling or predictive analysis

Multivariate modeling and predictive analysis | Linear mixed effects model were used to examine the relationship between skeletal muscle oxidative capacity and brain structural change.
